# Translation landscape of stress granules

**DOI:** 10.1126/sciadv.ady6859

**Published:** 2025-10-03

**Authors:** Yichun Wu, Xing Wang, Lingyu Meng, Zhizhao Liao, Wei Ji, Peipei Zhang, Jie Lin, Qiang Guo

**Affiliations:** ^1^ State Key Laboratory of Membrane Biology, Peking-Tsinghua Center for Life Sciences, Academy for Advanced Interdisciplinary Studies, School of Life Sciences, Peking University, Beijing 100871, China.; ^2^ Peking-Tsinghua Center for Life Sciences, Center for Quantitative Biology, Academy for Advanced Interdisciplinary Studies, Peking University, Beijing 100871, China.; ^3^ Academy for Advanced Interdisciplinary Studies, Peking University, Beijing 100871, China.; ^4^ Changping Laboratory, Beijing 102206, China.; ^5^ Institute of Biophysics, Chinese Academy of Sciences, Beijing, China.; ^6^ Key Laboratory for Neuroscience, Ministry of Education/National Health and Family Planning Commission, Department of Biochemistry and Molecular Biology, School of Basic Medical Sciences, Peking University Health Science Center, Beijing 100191, China.

## Abstract

Stress granules, cytoplasmic assemblies of RNA binding proteins and messenger RNAs formed during cellular stress, are implicated in translational control. However, their exact functions remain elusive. Here, we used cryogenic correlative light and electron microscopy to visualize stress granules in their native environment and reconstructed them in three dimensions using tomography. This approach provided the first quantitative and spatial analysis of the translational machinery within stress granules. Our findings suggest that stress granules have a limited impact on global translation regulation but serve to protect small ribosomal subunits and preinitiation complexes from degradation. Numerical simulations based on a phase-field model accurately reproduced the spatial distribution of ribosomal components inside and outside the stress granules, shedding light on the thermodynamic principles governing this process.

## Teaser

Cryo–electron tomography visualizes stress granules in situ, revealing their spatial interplay with translation machinery.

## INTRODUCTION

Stress granules (SGs) are dynamic, membrane-less organelles that appear in the cytoplasm when translation is compromised because of various stressors, including oxidative stress, temperature changes (*1*–*3*), or inhibition of translation factors (*4*). Biochemically, they contain stalled mRNAs, translation factors, and numerous proteins influencing the mRNA function (*5*–*7*). Notably, research has identified a link between mutations affecting their dynamics and the onset of various neurological diseases (*8*–*10*). However, the exact functional roles of SGs remain incompletely understood. Given that many SG components are translation modifiers and their formation coincides with reduced global translation, it is commonly assumed that they directly contribute to this repression (*11*). However, emerging evidence suggests that SGs may be dispensable for global translation shutdown (*4*, *12*), and translation levels may be comparable both within and outside them (*13*). Therefore, understanding the molecular architecture and cellular context of SGs is essential for a comprehensive understanding of their precise functions.

## RESULTS

As a first step to investigate SG formation and dynamics, we engineered a HeLa cell line stably expressing enhanced green fluorescent protein (EGFP)–tagged Ras GTPase (guanosine triphosphatase)–activating protein-binding protein 1 (G3BP1) and used sodium arsenite as an oxidative stressor. G3BP1 and its homolog G3BP2 play a critical role in establishing the percolation threshold for SG assembly, and the elimination of both proteins can prevent SG formation entirely (*14*). As expected, 500 μM sodium arsenite treatment efficiently induced the formation of SGs with distinct liquid-like properties (fig. S1, A to D). These SGs exhibited a tendency to coalesce into larger ones (fig. S1E and movie S1). Notably, the formation of SGs was reversible, with droplets gradually dissolving upon stress removal (fig. S1F and movie S2). Consistent with previous findings, the dynamics of SG formation appeared coupled to translation activity, as evidenced by the phosphorylation level of eukaryotic translation initiation factor 2 subunit α (eIF2α) (fig. S1G), a well-established marker of the integrated stress response (*15*), and by polysome profiling (fig. S1, H and I). To further characterize SGs, we vitrified the cells 1 hour after sodium arsenite treatment. Using our recently developed cryogenic correlated light, ion, and electron microscopy technique (*16*), we prepared SG-containing cell samples and visualized them using cellular cryo–electron tomography. This approach enabled us to effectively capture SGs and visualize their cellular context at a high resolution.

**Fig. 1.**
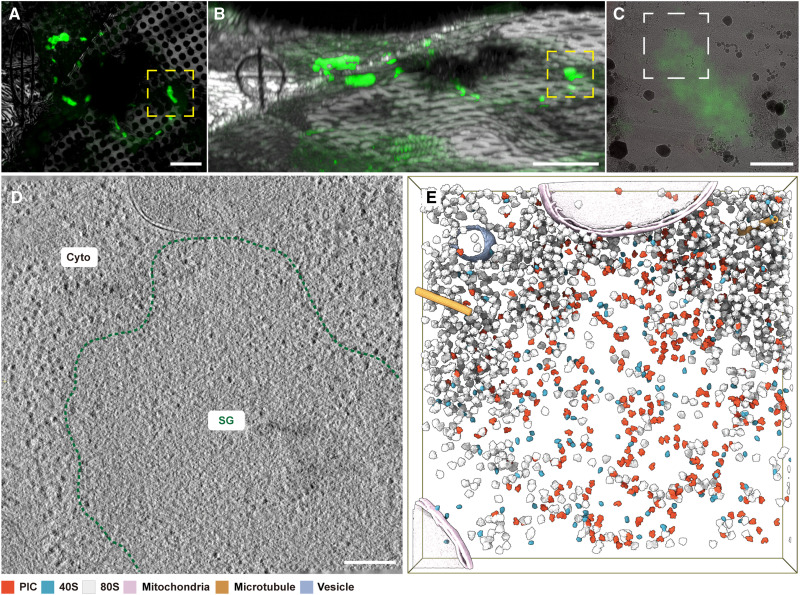
In situ analysis of SGs within HeLa cells. (**A**) The cryo–light microscopy image shows HeLa cells grown on an electron microscopy grid. SGs are labeled with G3BP1-EGFP (green). The dashed yellow box highlights the SG chosen for lamella preparation. (**B**) An FIB image of the same region as (A). The light microscopy image stack is transformed to align with the FIB image orientation and overlaid to define the milling position. The dashed yellow box indicates the corresponding SG in (A). (**C**) Superimposed image of the prepared lamella obtained using light microscopy and TEM corresponding to the region marked by the dashed yellow box in (B). (**D**) Tomographic slice of the HeLa cell corresponding to the white boxed region in (C). The boundary of the SG is outlined by the green dashed line, and the regions of the cytoplasm and SG are labeled in the image. (**E**) 3D rendering of the tomogram of (D). Ribosomal components, including 40S ribosomes (blue), PICs (orange), and 80S ribosomes (gray), are computationally identified and placed back into their original locations and orientations. Microtubules (yellow), mitochondria (pink), and endoplasmic reticulum (ER) vesicles (purple) are computationally segmented and shown as surface models. Scale bars, 10 μm in (A) and (B), 1 μm in (C), and 200 nm in (D).

As anticipated, the SGs themselves appeared featureless under transmission electron microscopy (TEM) (*17*). The correlative EGFP signal guided the tilt series collection for tomographic reconstruction (Fig. 1, A to C). The resulting three-dimensional (3D) data revealed that SGs form electron-dense regions with irregular shapes (Fig. 1, D and E), partially consistent with previous observations using conventional TEM (*12*, *17*). The captured granules were at their late stage, with diameters exceeding 1 μm. We observed few microtubules surrounding them (Fig. 1E), contradicting the hypothesis that small granules are transported together by a dynein-based mechanism (*18*, *19*). While a previous work suggested the involvement of membrane-bound organelles in SG dynamics (*20*), our tomograms showed limited membrane-based structures in close contact with them. We remark that the observations at this late time point may not fully represent the complete sequences of events.

Preliminary inspection of reconstructed tomograms suggested a generally homogeneous ultrastructure for SGs (Fig. 1 and fig. S2, A and B). The absence of readily discernible scaffolds or cores, previously reported in the literature (*21*), is likely attributable to the limitations of our spatial resolution. Sparsely and randomly distributed molecules with a diameter of about 30 nm were detected within the electron-dense regions, reminiscent of ribosomes (Fig. 1, D and E, and fig. S2, A and B). Compared to the surrounding cytosolic areas, these molecules exhibited greater size diversity and a substantially lower concentration (Fig. 1, D and E, and fig. S2, A to C). To address the ongoing controversy surrounding the translation activity within and external to SGs (*13*), we pooled all the putative ribosome particles together in the stress-induced cells for a comprehensive structural analysis.

**Fig. 2.**
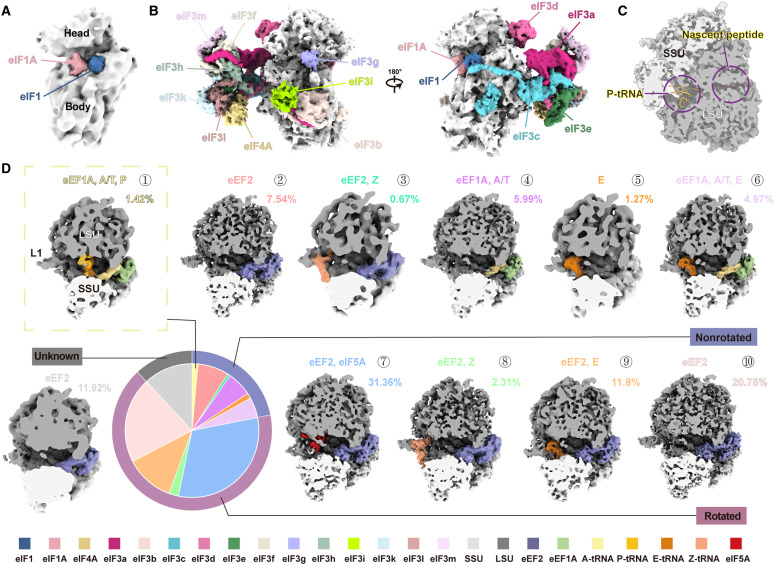
Structural analysis of ribosomal components under stress conditions. (**A**) The density map of the 40S ribosome is displayed in a solid surface representation. Putative densities corresponding to initiation factors eIF1 and eIF1A are highlighted in blue and pink, respectively. (**B**) The density map of PIC is shown in a solid surface with two different views. Densities corresponding to initiation factors (eIF1, eIF1A, eIF4A, and subunits of eIF3) are colored as indicated. (**C**) This cross-sectional view of the density map shows the overall structure of 80S ribosomes directly reconstructed under stress condition, revealing an empty P-site and nascent peptide tunnel, indicating a nontranslating ribosome. The 40S small subunit (SSU) is colored light gray, and the 60S large subunit (LSU) is colored dark gray. (**D**) All 80S ribosomes from stressed cells were pooled and classified on the basis of the occupancy of tRNAs (A-, P-, E-, and Z-sites colored light yellow, dark yellow, orange, and soft orange, respectively), translation factors (eEF1A in green, eEF2 in purple, and eIF5A in red), and the ribosome “ratcheting” state (rotated, nonrotated, and unknown). This classification resulted in 11 distinct structures displayed in a solid surface representation. The pie chart summarizes the occupancy of tRNAs and factors, the ratcheting state, and the proportion of each identified conformation. The structure corresponding to actively translating ribosomes is highlighted with a dashed box. Source numerical data are available in dataset S1.

Our initial structural analysis revealed four distinct ribosomal components: the preinitiation complex (PIC), the 40S small subunit, the 60S large subunit, and the 80S ribosome (Figs. 1E and 2 and figs. S3 and S4). By docking previously resolved structures of in vitro–purified samples, we characterized the composition and status of these translation machineries (figs. S3 and S4). Detailed structural analysis revealed that the 40S subunit displayed bound densities near the decoding center (Fig. 2A). Although we could not confirm the protein identities because of limited resolution, the position and shape suggest that eIF1 and eIF1A might contribute to these densities, stabilizing an open 40S conformation for the binding of following initiation factors (*22*). At a resolution of 10 Å, we elucidated the composition of PIC (Fig. 2B). Densities for eIF1, eIF1A, and all eIF3 subunits, except 3j, were clearly resolved with high occupancy. However, the eIF2-GTP-tRNA ternary complex was completely absent, indicating a stalled preinitiation state (*23*), which aligns with immunostaining data (*3*, *24*) and the observation of phosphorylated eIF2α (fig. S1G) that blocks the formation of a ternary complex (*25*).

Although the translation is known to substantially slow down, if not totally shut down after stress, most ribosome particles are in the 80S form (fig. S1J). Direct reconstruction of these 80S particles showed them with an empty P-site and nascent peptide tunnel, resembling translationally idle ribosomes (Fig. 2C) (*26*, *27*). To uncover the overall translation elongation cycle under stress, we used an exhaustive classification approach to analyze the conformations of 80S ribosomes (figs. S3 and S4). Notably, among the resulting 11 80S ribosome conformations, only one conformation (constituting merely 1.4% of the total 80S population) exhibited the P-site tRNA binding conformation indicative of active translation elongation (Fig. 2D, yellow box). Conversely, more than 85% of the population displayed eEF2 occupancy at the A-site alongside a vacant P-site, a configuration characteristic of the translationally inactive state (Fig. 2D). In accordance with these findings, we barely detected polysomes using either the neighboring analysis method (fig. S5A) (*28*) or biochemical assays (fig. S1J). Intriguingly, we unveiled a previously unidentified ribosome dimer mediated by Ebp1 and the ribosomal RNA (rRNA) expansion segment ES27L (fig. S5C), suggesting a potential “hibernating” state, as both ribosomal exit tunnels appear positioned in close proximity, potentially hindering nascent peptide exiting. It is noteworthy that the interaction between Ebp1 and ES27L has been well characterized in inactive 80S monosomes (fig. S5C) (*29*).

To substantiate the role of oxidative stress treatment in the observed global translational shutdown, independent of G3BP1-EGFP expression, we used cellular tomography on the same construct under nonstressed conditions. This approach captured PIC with and without a ternary complex (fig. S6A). Notably, the 80S ribosome translation elongation landscape and polysome distribution in our system closely resembled those observed in other recently resolved eukaryotic cells (figs. S5B and S6, B and C) (*30*, *31*).

**Fig. 3.**
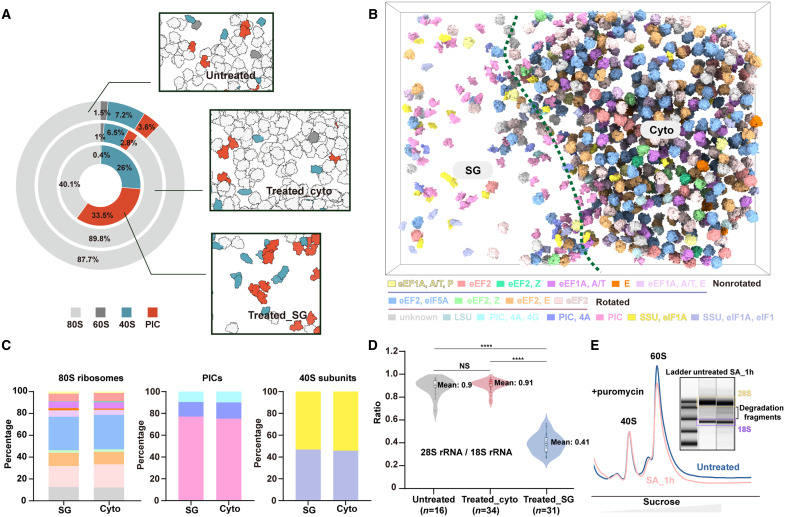
Topography of ribosomal components. (**A**) The relative abundance of 40S subunits (blue), PICs (orange), 60S subunits (dark gray), and 80S ribosomes (light gray) within and outside SGs of HeLa cells treated with sodium arsenite is depicted in a donut chart. The chart also includes results from the cytosolic region of control cells without stress. Representative 3D renderings of these cellular regions are shown alongside the corresponding data points in the chart. (**B**) Tomographic 3D rendering in the SG boundary region. The boundary of the SG was indicated by the green dashed line. Ribosomal components were placed back in their original orientations and locations with distinct colors, including 80S ribosomes (11 states), 60S subunits, 40S subunits (two states), and PICs (three states). (**C**) The bar charts depict the distribution of the identified ribosomal conformations (80S ribosomes, PICs, and 40S subunits) within and outside SG. The high degree of similarity in distribution between the two locations is further supported by a statistically significant Kolmogorov-Smirnov test (80S ribosomes: *P* = 0.997; PICs: *P* = 0.999; 40S subunits: *P* = 0.999). (**D**) A violin plot describes the changes of the 28S rRNA–to–18S rRNA ratio within SGs and the cytoplasm after sodium arsenite treatment, as well as in the cytoplasm of control HeLa cells. Statistical methods: *t* test and Mann-Whitney *U* test were used (****P < 0.0001). (**E**) Analysis of ribosomal component changes before and after treatment by ribosome profiling with puromycin and RNA integrity analysis. Source numerical data are available in dataset S2.

The SGs are traditionally viewed as pools for stalled PICs with few mature 60S components (*17*, *24*). By sorting ribosomes on the basis of their subcellular locations, we observed distinct spatial distributions for these ribosomal components (Fig. 3A and fig. S2, A to C). We found that SGs specifically enrich 40S small subunits and PICs. Unexpectedly, they still contain a notable amount of 80S, inconsistent with previous works (*5*, *24*, *32*). For the remaining part of the cytosol, the distribution of these different ribosomal components is nearly identical to that of untreated control cells (Fig. 3A and fig. S2, C and D). Notably, even though the local concentration of ribosomal components undergoes alteration upon stress induction, their spatial arrangement within SGs appears to be random (fig. S2, E to G). The aforementioned homogeneous character of these droplets is further corroborated by this observation.

By further sorting the 80S ribosome conformations inside and outside SGs, we found that the conformations displayed unexpectedly similar distributions regardless of the subcellular context (Fig. 3, B and C). This implies that the translation elongation kinetics are similar both within and outside SGs, even though translation is generally suppressed under stress conditions. This is consistent with the observation that the entire translation cycle can occur within these granules (*13*). This observation held true for the PIC and 40S subunit as well, albeit limited classes were identified (Fig. 3C). These findings suggest that SGs alter the spatial distribution of translation machinery, specifically enriching PICs and 40S subunits, while the microenvironment has a minimal impact on their conformational states.

We remark that SGs exhibit a significantly higher concentration of small subunit–containing ribosomal components (i.e., the sum of 40S, PIC, and 80S) than that of large subunit–containing ones (i.e., the sum of 60S and 80S) (Fig. 3A). The tightly regulated biogenesis of large and small ribosomal subunits ensures their equal stoichiometry within the cell (*33*). The imbalance we observed here (Fig. 3D), considering the substantial total volume of SGs within each cell (fig. S1), may play an important role in translation regulation.

This imbalance could arise from either differences in subunit biogenesis or preferential degradation of one subunit over the other. To explore this, we performed RNA sequencing to analyze the expression levels of ribosome biogenesis–related genes and ribosomal proteins before and after sodium arsenite treatment (fig. S7A). Notably, no significant changes were observed in the expression of these genes, suggesting that the biogenesis rates of both subunits remain comparable under stress conditions. This finding raises a compelling hypothesis: Under stress, a substantial portion of large ribosomal subunits may be selectively degraded. To evaluate the relative decay rates of large and small ribosomal subunits, we conducted ribosome profiling and assessed rRNA integrity at multiple time points (Fig. 3E). Time-course analysis revealed that this degradation coincides with SG formation, translational repression, and polysome disassembly (figs. S1H and S7B and movie S1).

We therefore speculate a role of SGs in protecting ribosome small subunits from degradation: During stress, most translational machineries enter the idle state, with the A-site occupied to prevent RQC (ribosome-associated quality control) pathway activation (Fig. 2, C and D) (*34*). In cases where 80S ribosomes split, the small subunits and PICs get protected within SGs, while the exposed large subunits become vulnerable to degradation.

To further validate this role of SGs, we conducted experiments using a cell line in which SG formation is abolished because of the knockout of two essential SG genes, G3BP1 and G3BP2 (fig. S8A). Under sodium arsenite–induced stress, phosphorylation of eIF2α led to stalled translation initiation and the accumulation of ternary complex–deficient PICs, which remained unaffected by the absence of SG formation in the double-knockout (DKO) cells (fig. S8, B and C). This observation suggests that while SG formation coincides with reduced global translation, it does not directly contribute to this repression. Consistent with this, the analysis of translating 80S ribosomes revealed that the majority remained in a translation-stalled state in sodium arsenite–treated DKO cells (fig. S8, D and E). In the absence of SGs under sodium arsenite stress, the distribution of ribosomal components closely resembled that of the cytoplasm in untreated cells, as well as the cytoplasm of cells treated with sodium arsenite (fig. S8, F and G). This indicates a loss of the protective function for small ribosomal subunits in the absence of SGs.

**Fig. 4.**
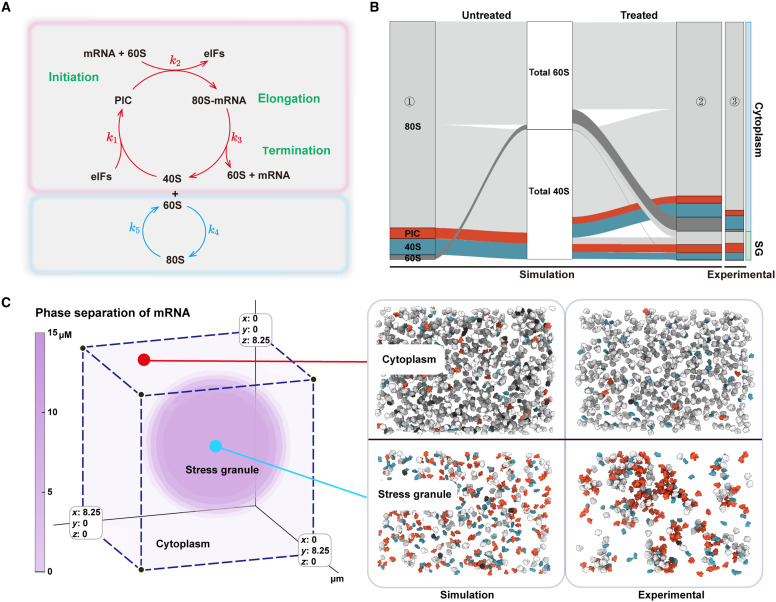
Phase-based model incorporating chemical reactions recapitulating experimental data. (**A**) Schematic of simulated reactions of ribosomal components. (**B**) Visualization of simulated ribosomal component dynamics using a Sankey diagram. Panel 1 depicts the baseline concentrations of ribosomal components in untreated cells. Panel 2 showcases the simulated concentrations of these components specifically located within the cytoplasm (light blue) and SGs (light green) after 1 hour of sodium arsenite treatment. The total small subunit–containing ribosomal components (i.e., the sum of 40S, PIC, and 80S) and large subunit–containing ribosomal components (i.e., the sum of 60S and 80S) remain constant before and after treatment, as illustrated in the middle white panel. Panel 3 exhibits the corresponding experimental data for comparison. The ribosomal components include 80S ribosomes (light gray), PICs (orange), 40S subunits (blue), and 60S subunits (dark gray). (**C**) Simulated phase-based model depicting SG formation following sodium arsenite treatment. The presented 3D model illustrates mRNA phase separation, with darker colors (purple) indicating higher concentrations. The concentration and length are represented in μM and μm, respectively. The comparison of simulated and experimental particle distributions within and outside SG is shown on the right. Ribosomal components, including 80S ribosomes (light gray), PICs (orange), 40S subunits (blue), and 60S subunits (dark gray), are placed back according to their locations and orientations within different regions (cytoplasm and SG). For the simulation, particles are generated from a continuous concentration field derived from the phase-field simulations and assigned random orientations. Source numerical data are available in dataset S3.

We sought to quantitatively recapitulate our experimental observations using a simple physics-based model. Intriguingly, the formation of SGs under oxidative stress and the compositions of ribosomal subunits within different compartments were quantitively recapitulated by a phase-field model with conserved ribosomal subunits incorporating chemical reactions (Fig. 4) (*35*–*38*). When the stress is absent, the ribosomal subunits are mainly involved in a translation loop, in which each step is energy-consuming and does not satisfy detailed balance such that the reverse reactions are negligible. Meanwhile, we also introduced an equilibrium reaction in which the free 40S and 60S subunits assemble to inactive 80S ribosomes reversibly (Fig. 4A) (*27*). Previous experiments suggested that the availability of free mRNAs is crucial to induce SGs (*39*, *40*). Therefore, we assumed that free mRNAs can form condensates through attraction with the key protein G3BP1, as confirmed experimentally recently (*41*). The effects of oxidative stress include a reduced binding rate of PIC and 60S on mRNA because of missing a ternary complex (i.e., a reduced $k_{2}$ in the translation loop), which generates more free mRNAs. We also assumed that the assembly rate of inactive 80S from 40S and 60S increases under oxidative stress so that inactive 80S can be accumulated (*26*). We simulated the model in three dimensions and confirmed the induction of SGs by oxidative stress (Fig. 4, B and C). Intriguingly, with a weak attraction between 40S/PIC and G3BP1 (*42*) and a repulsion between 60S/80S and G3BP1, the simulated concentrations of all ribosomal components, except 60S in the cytoplasm, quantitatively match the experimental measurements (Fig. 4B). These results suggest that the enrichment of 40S/PIC and the exclusion of 60S/80S in SGs may be driven by thermodynamics. Meanwhile, the difference in 60S concentration in the cytoplasm between simulation and experiment suggests that the degradation of 60S subunits is nonnegligible after the formation of SGs.

To determine whether the observed phenomenon is specific to sodium arsenite treatment or represents a general feature of SGs, we also investigated SGs induced by heat shock (fig. S9). HeLa cells expressing G3BP1-EGFP were subjected to heat shock at 43°C for 1 hour, leading to the formation of micrometer-scale SGs. Using correlative light and electron microscopy, we obtained high-resolution ultrastructural details of these granules in situ (fig. S9, A to C). The overall architecture of heat shock–induced SGs closely resembled those formed under sodium arsenite treatment (fig. S9C). Subsequent subtomogram averaging analysis revealed a similar spatial distribution of ribosomal components in heat shock–treated cells compared to sodium arsenite–treated cells, with subtle differences in their relative proportions (fig. S9, D to J). These differences likely reflect the milder stress conditions induced by heat shock, as evidenced by lower phosphorylation levels of eIF2α and a slightly higher proportion of translating 80S ribosomes (fig. S9, K to M). Despite these variations, a significant imbalance in ribosomal subunits was also observed in heat shock–treated cells (fig. S9J).

Collectively, these findings suggest that the selective enrichment of ribosomal components and the imbalance between subunits are conserved features of SGs under different stress conditions. This supports the notion that SGs play a conserved role during cellular stress responses.

## DISCUSSION

The formation of SGs coincides with a suppression of translation initiation. This, coupled with the selective accumulation of small ribosomal subunits and nontranslating mRNAs, suggests that SGs may play a regulatory role in repressing translation (*32*). However, recent single-molecule studies have challenged this paradigm by demonstrating the presence of ongoing translation within SGs (*13*). The 80S ribosome conformation landscape we found, despite the marked reduction in translational activity in stressed cells, strongly suggests that the translation elongation and inhibition proceed identically inside and outside SGs. This finding is further corroborated by prior studies, demonstrating that translation remains inhibited by arsenite stress even in cells lacking G3BP, a protein crucial for SG formation (*43*). The observed inhibition of translation likely arises directly from globally impaired translation initiation. This aligns with the previously documented phosphorylation of eIF2α (*25*), which leads to an accumulation of stalled PICs devoid of ternary complexes, as confirmed structurally in this work. Depletion of ternary complexes restricts translation initiation primarily to noncanonical pathways for a select group of genes (*44*). Thus, our findings suggest that stalling translation initiation is a key mechanism for translation inhibition, while SG formation appears to play a limited role.

Our numerical simulations support the picture that the formation of SGs is due to the accumulation of free mRNAs. The slowdown of translation also frees more ribosomal subunits, leading to the accumulation of inactive 80S ribosomes both inside and outside SGs. Our simulations further suggest that the enrichment of 40S/PIC and the exclusion of 60S/80S in SGs may have a thermodynamic origin. We cannot exclude the effects of energy-consuming reactions in the formation of SGs (*45*), and it will be interesting to disentangle the effects of active processes from passive ones.

While our data collectively show that SG formation has a limited impact on translational elongation activity, translation inhibition still occurs even in the absence of SGs, as observed in DKO cells. This raises an interesting question: What is the potential role of SGs? Ribosomes are renowned for their remarkable longevity. However, disassembled small and large subunits are prone to degradation (*34*). The degradation of ribosomes under oxidative stress conditions has been observed at both the protein and RNA levels (*46*). Given the susceptibility of disassembled ribosomal subunits to degradation under oxidative stress conditions, our analysis suggests that the sequestration of small ribosomal subunits in SGs may serve as a protective mechanism against degradation, while large subunits are more likely to undergo degradation. As the platform for mRNA recognition and initiation factor binding, small subunits are critical for regulating translation initiation. The colocalization of 40S subunits and PICs with mRNA within SGs enables rapid translation reactivation upon stress removal. Concurrently, the degradation of 60S subunits could be counterbalanced by two compensatory mechanisms: (i) de novo biogenesis, which is rapidly up-regulated poststress (*47*), and (ii) recycling of preexisting 60S subunits from nontranslating 80S monosomes. Although this results in a transient stoichiometric imbalance during stress, the combined effects of SG-protected 40S/PICs (enabling immediate initiation) and efficient 60S replenishment allow cells to restore translation capacity swiftly.

## MATERIALS AND METHODS

### Plasmid construction

Total RNA was extracted from human embryonic kidney (HEK) 293F cells using the RNAprep Pure Cell Kit (Tiangen Biotech, DP430) following the manufacturer’s protocol. The extracted RNA was reverse transcribed into cDNA using the TransGen AT411 reserve transcription kit. To isolate the coding sequence of G3BP1, specific primers were designed: forward primer, 5′-CTCGAGCTTCGAATTCATGGTGATGGAGAAGCCTAG-3′, and reverse primer, 5′-GGCACCCCTTGCTCACCATGAATTCCTGCCGTGGCGCAAGC-3′.

The amplified G3BP1 cDNA was then cloned into the lentiviral pLVX-EGFP transfer plasmid via homologous recombination using the ClonExpress II One Step Cloning Kit (C112-01) following the manufacturer’s protocol. For lentiviral production, the pMD2.G envelope plasmid and the psPAX2 packaging plasmid were also prepared. All constructs were confirmed by Sanger sequencing to ensure accuracy.

### Construction of a stable G3BP1-EGFP–expressing HeLa cell line

HEK293T cells were cotransfected with three plasmids—the pLVX-EGFP transfer plasmid encoding G3BP1-EGFP, the pMD2.G envelope plasmid, and the psPAX2 packaging plasmid—using a polyethylenimine-mediated transfection protocol, maintaining a polyethylenimine-to-plasmid ratio of 3:1. After 48 hours of incubation, the supernatant containing lentivirus particles was collected and centrifuged at 3000 rpm for 5 min to remove cellular debris. The resulting lentivirus was used to infect HeLa cells, with polybrene added to the culture medium at a final concentration of 8 μg/ml to enhance transduction efficiency. Following a 48-hour infection period, the transduced HeLa cells were selected in the presence of puromycin (1 μg/ml). The resulting stable HeLa-G3BP1-EGFP cell line was maintained in Dulbecco’s modified Eagle’s medium supplemented with 10% fetal bovine serum under a humidified atmosphere at 37°C with 5% CO_2_.

To induce the formation of SGs, HeLa-G3BP1-EGFP cells were treated with 0.5 mM sodium arsenite (Innochem, A25410) for 1 hour. This treatment led to the formation of SGs larger than 1 μm in diameter, suitable for subsequent experiments analysis.

### Immunofluorescence

Sterile 14-mm glass coverslips were placed in 24-well plates and coated with poly-d-lysine (1 mg/ml) for 4 hours, followed by three washes with deionized water. HeLa-G3BP1-EGFP cells were seeded onto the coverslips and incubated for 12 hours. The cells were then washed twice with phosphate-buffered saline (PBS) and fixed with 4% paraformaldehyde at room temperature for 20 min. After fixation, the cells were washed three times with PBS and permeabilized with a solution containing PBS, 0.1% Tween 20, and 0.2% Triton X-100 for 15 min at 4°C. Following permeabilization, the cells were washed three times with PBS containing 0.1% Tween 20 (PBST) and blocked with 5% bovine serum albumin in PBST for at least 1 hour at room temperature. Subsequently, a primary antibody (TIA-1, Santa Cruz Biotechnology, sc-166247) was added and incubated with the cells overnight at 4°C. On the following day, the cells were washed three times with PBST and incubated with a fluorophore-conjugated secondary antibody [Goat anti-Mouse IgG (H + L) Highly Cross-Adsorbed Secondary Antibody, Alexa Fluor Plus 647, Invitrogen, A32728] for 1 hour at room temperature. After three final washes with PBST, the coverslips were mounted with an antifade mounting medium and stored at 4°C until visualization.

### Sucrose gradient and ribosome profiling

For each experimental condition, cells were cultured in three 10-cm dishes to 70 to 80% confluence and treated with cycloheximide (CHX; 100 μg/ml; Aladdin, C112766) at 37°C for 5 min. Cells were collected in 500 μl of scraping buffer containing CHX (100 μg/ml), a protease inhibitor (M5, MF-1821), and Superase In (20 U/ml; Invitrogen, AM2694). The suspension was centrifuged at 500*g* for 5 min. Cells were lysed in 500 μl of lysis buffer [5 mM tris-HCl (pH 7.5), 100 mM KCl, 5 mM MgCl_2_, protease inhibitors, 1 mM dithiothreitol, CHX (200 μg/ml), 2% Triton X-100, and Superase In (20 U/ml)] on ice for 10 min. The lysate was centrifuged at 13,000 rpm for 10 min at 4°C to remove cellular debris. The clarified lysate was loaded onto a 10 to 45% sucrose gradient prepared in a buffer containing 20 mM Hepes-KOH (pH 7.4), 100 mM KCl, 5 mM MgCl_2_, protease inhibitors, 1 mM dithiothreitol, and CHX (200 μg/ml). Ultracentrifugation was performed at 30,000 rpm for 3 hours at 4°C. The sucrose gradient was fractionated using a Biocomp gradient fractionation system for ribosome profiling analysis.

To dissociate 80S ribosomes into 60S and 40S subunits, cells were washed twice with PBS containing CHX (100 μg/ml). The washed cells were then lysed in a buffer containing 500 mM KCl, 2 mM MgCl_2_, 15 mM Hepes (pH 7.4), 2 mM puromycin, and 1% Triton X-100. Lysates were centrifuged at 12,000*g* for 5 min at 4°C to remove debris and layered onto a 10 to 30% sucrose gradient prepared in a buffer containing 500 mM KCl, 2 mM MgCl_2_, 15 mM Hepes (pH 7.4), and diethyl pyrocarbonate–treated water. Ultracentrifugation was performed at 39,000 rpm for 2.5 hours at 4°C to separate ribosomal components. Fractionation of the sucrose gradient was performed using a Biocomp gradient fractionation system to analyze ribosomal subunits.

### Fluorescence recovery after photobleaching analysis

HeLa cells stably expressing G3BP1-EGFP were seeded into 35-mm glass-bottom dishes and allowed to adhere overnight at 37°C in a humidified incubator with 5% CO_2_. SGs were induced by treating the cells with 0.5 mM sodium arsenite for 1 hour. For fluorescence recovery after photobleaching analysis, several circle regions of interest ~3.5 μm in diameter, located within the cytoplasm SGs, were selected. Photobleaching was performed using a Nikon A1RSi+ laser scanning microscope equipped with a 488-nm laser and a 100× oil-immersion objective. The laser was set to 50% of the available laser power, and bleaching was performed at a scanning speed of 1. Fluorescence recovery within the bleached region of interest was recorded every second for 2 min postbleaching. Following the above method, data obtained from a total of 13 distinct SGs were used to construct the average fluorescence recovery curve.

### Western blot analysis

HeLa-G3BP1-EGFP cells, U2OS cells, and DKO G3BP1/G3BP2 U2OS cells were used for Western blot analysis. The harvested cell pellets were lysed in a buffer containing 20 mM tris (pH 7.5), 150 mM NaCl, and 1% Triton X-100 and supplemented with sodium pyrophosphate, β-glycerophosphate, EDTA, Na_3_VO_4_, leupeptin, protease, and phosphatase inhibitors. The lysates were centrifuged at 12,000 rpm for 5 min at 4°C to remove cellular debris. The resulting supernatant was subjected to SDS–polyacrylamide gel electrophoresis to separate proteins, which were then examined using antibodies targeting eIF2α (Proteintech, 11170-1-AP), phospho-eIF2α (Cell Signaling Technology, no. 3398), GAPDH (glyceraldehyde-3-phosphate dehydrogenase; Abcam, AB8245), G3BP1 (Proteintech, 13057-2-AP), and G3BP2 (Proteintech, 16276-1-AP).

### Live-cell imaging

Live-cell imaging was performed using a Carl Zeiss LSM880 Confocal Microscope equipped with a Plan-Apochromat 63×/1.4 Oil DIC M27 objective, a 488-nm laser, and a live-cell station maintaining physiological conditions at 37°C and 5% CO_2_. Imaging parameters were set as follows: interval of 2 min, fluorescence autofocus mode, resolution of 512 by 512 pixels, frame scan mode, line step of 1, scan speed of 7, averaging number of 2 (mode: line; method: mean; 16-bit depth), 488-nm laser intensity at 2.0%, pinhole size of 90.2, and detector gain set at 650. Initially, cells of interest were imaged to establish baseline conditions. Cells were then treated with 0.5 mM sodium arsenite. Immediately following this treatment, time-lapse imaging was initiated, capturing images every 2 min for 1 hour. After treatment, the sodium arsenite–containing medium was replaced, and cells were washed three times with fresh medium to ensure the complete removal of the drug. Subsequently, normal medium was reintroduced, and the recovery was monitored through live-cell imaging for up to 6.5 hours.

### Cryo–electron tomography sample vitrification

Electron microscopy grids (Beijing Xinxing Bairui Technology, T10012Au; QUANTIFOIL, Au 200 mesh, R2/1) were subjected to carbon deposition (thickness of ~20 nm) to improve their mechanical stability and provide a suitable surface for cell adhesion. The grids were then glow discharged for 1 min with H_2_/O_2_ using the Gatan Plasma System (Model 950 Advanced Plasma System) to increase hydrophilicity. After ultraviolet sterilization for 30 min, the grids were placed in a six-well plate, seeded with cells, and incubated overnight at 37°C in a humidified atmosphere containing 5% CO_2_ to ensure cell attachment and growth. Following treatment (0.5 mM sodium arsenite for 1 hour or heat shock at 43°C for 1 hour), the grids were rapidly vitrified in liquid ethane using a plunge freezer (Vitrobot Mark IV, FEI). The blotting time was set to 10 s with a blot force of 10. Vitrified samples were stored in liquid nitrogen until further processing for focused ion beam (FIB) milling.

### Correlative light, ion, and electron microscopy

Using the correlative light, ion, and electron microscopy approach developed by us (*16*), regions containing SGs were identified and screened by scanning electron microscopy and confocal imaging. The sample was then sputter coated with a conductive protective layer to minimize charging effects and surface damage during milling. A cross-shaped reference pattern was etched adjacent to the selected region for accurate localization. The sample stage was subsequently moved to the confocal microscope, where stacks of images from the selected region were captured, followed by 3D reconstruction using ImageJ. The distance between the target SG and the center of the reference pattern was measured by projecting the confocal image in alignment with the scanning electron microscopy image, which was used to determine the exact location of the SG on the FIB image. Following this, a lamella of ~1.3 μm in thickness was fabricated at the determined position. The lamella was then reimaged using a confocal microscope, and the SG position relative to the lamella boundaries was determined. After successive thinning and confocal imaging, precision trimming of the lamella resulted in a final lamella of ~150 nm in thickness containing the SG region.

### Tilt series acquisition

Data acquisition was performed on Titan Krios microscopes (Thermo Fisher Scientific) with a K2 (Gatan) or a post-GIF K3 camera (Gatan). For the sodium arsenite–induced SG samples, initially, the data were collected at a lower magnification (pixel size: 4.3 Å) with a K2 camera to get an overview of the SGs. Following this, the K3 camera was used to capture 67 tilt series (pixel size: 1.37 Å) using the parallel cryo–electron tomography (PACE-tomo) method (*48*), including 33 from the SG regions and 34 from the cytoplasmic periphery of the SG. For the untreated control cells, tilt series were collected at two pixel sizes: 1.37 Å (43 tomograms) and 2.67 Å (40 tomograms). Similarly, heat shock–treated cells were imaged at 1.37 Å (33 tomograms), with 10 tomograms from SG regions and 23 from the cytoplasm. Tilt series were acquired with SerialEM software (*49*) using a dose-symmetric tilt scheme (*50*), with 2° increments ranging from +60° to −40° and a defocus range of −2 to −5 μm. Data were recorded in dose fractionation mode, with a cumulative electron dose of ~110 e^−^/Å^2^ across each tilt series.

### Tomogram reconstruction and segmentation

The TOM toolbox (*51*) and TOMOMAN (*52*) were used as the general platform for image processing. Image stacks of each tilt images were aligned using Motioncor2 (*53*). The resulting tilt series were then precisely aligned through the patch tracking method and reconstructed using the weighted back-projection algorithm within IMOD/4.11.0 software (*54*). For visualization, the scale of the tomograms was adjusted with a binning factor of 4 or 6, and a deconvolution filter was applied to enhance contrast (*55*). Automatic segmentation of all membranes was initially performed using MemBrainV2 (*56*), followed by manual refinement using Amira (Thermo Fisher Scientific). The 3D renderings of the segmented tomograms were generated using ChimeraX/1.9 (*57*).

### Subtomogram averaging and classification

Template matching was conducted using STOPGAP (*58*) with low-pass–filtered maps of 40S and 80S ribosomal components as reference. After manual screening, the resulting coordinates (treated 40S subunit: 4400; PIC: 4507; 80S ribosome: 24,873) were then used for extracting subtomograms by Warp/1.1.0 BETA (*55*), which also conducted CTF estimation and tomogram reconstruction. The pixel size for these subtomograms was set at 2.74 Å (bin2).

After excluding the nonribosomal particles through classification, the remaining subtomograms were aligned using RELION/3.1 (*59*), and then the coordinates and the corresponding half-map were transferred to M/1.0.9 (*60*) for further processing. The refinement process in M continued for five iterations, and the final refinement was performed using RELION, culminating in a final resolution of 11, 10, and 6.3 Å for the sodium arsenite–treated ribosomal particles (40S, PIC, and 80S).

For the classification of 80S ribosomes, the well-aligned particles were first classified on the basis of the ribosomal ratcheting status using a tight 80S mask. Within each ratcheting status, classification was performed focusing on the three tRNA sites (A-, P-, and E-sites) with a designed mask. For 40S subunits and PICs, eIF1-, eIF4-, and ternary complex–focused masks were used. Refinement was performed for each resulting structure, and the resolution was estimated on the basis of the gold-standard Fourier shell correlation.

### Ribosomal component distribution analysis

For the topological analysis of polysomes, NEMO-TOC was performed on the two datasets (treated and untreated cells) (*28*). The concentration of ribosomal components was calculated using the formula *N*/(*N*_A_ * *V*_tomo_), where *N* represents the number of ribosomal components, *N*_A_ is the Avogadro constant, and *V*_tomo_ is the volume of the tomogram. The volume of each tomogram were measured in Amira by excluding the regions occupied by organelles, such as the endoplasmic reticulum (ER), mitochondria, and nucleus. Statistical analysis was subsequently performed using Origin software. Student’s *t* tests were used to assess the significance of differences between normally distributed data, while the Mann-Whitney *U* test was applied to skewed distribution data. Regarding the analysis of spatial distribution, for each scalar wave number q , the corresponding structure factor of one ribosomal component is calculated as

$$\begin{array}{c} S\left(q\right)=\sum_{m=1}^{N_{\text{tomo}}}\left[\frac{1}{N_{m}}\sum_{j=1}^{N_{m}}\sum_{k=1}^{N_{m}}\left\langle e^{-iq\cdot \left(r_{j}-r_{k}\right)}\right\rangle \right]/N_{\text{tomo}} \end{array}$$

Here, $N_{\text{tomo}}$ is the total number of tomograms for calculation, and $N_{m}$ is the particle number of the component in the m th tomogram. To obtain the structure factor for a scalar q , we generate vectors q that are uniformly distributed in the spherical coordinate with ∣q∣=q and calculate the average among all vectors q as ⟨…⟩ in the equation. $r_{j}$ is the position vector of the j th particle with the unit pixel (2.74 Å).

### RNA integrity analysis

Total RNA was extracted from both untreated and sodium arsenite–treated cells using TRIzol reagent (Invitrogen, 15596026) according to the manufacturer’s instructions. Following extraction, 10 μg of RNA from each sample was analyzed for quality and integrity, with a focus on the 18S and 28S rRNA markers, using the Agilent 2200 TapeStation system (Agilent Technologies). The system generated an electropherogram profile and gel-like imaging, providing a detailed visual assessment of the RNA quality and integrity.

### Numerical simulation

We used a phase-field model, comprising chemical reactions and diffusion to describe the dynamics of the concentration fields of 40S, eIFs, PIC, 60S, mRNA, G3BP1, 80S (inactive ribosomes without mRNA), and n×80S−mRNA (active polysomes with n ribosomes bound to a single mRNA). The model assumed that these components, except G3BP1, engage in a series of chemical reactions

$$\begin{array}{c} 40S+\text{eIFs}\;\overset{\;k_{1}\;}{\to }\;\text{PIC} \end{array}$$

$$\begin{array}{c} \text{PIC}+\text{mRNA}+60S\;\overset{\;k_{2}\;}{\to }\;1\times 80S-\text{mRNA}+\text{eIFs} \end{array}$$

$$\begin{array}{c} \begin{array}{c} \text{PIC}+60S+\left(n-1\right)\times 80S-\text{mRNA}\;\overset{\;k_{2}\;}{\to }\;n\times 80S-\text{mRNA}+\text{eIFs} \end{array} \end{array}$$

$$\begin{array}{c} n\times 80S-\text{mRNA}\;\overset{\;k_{3}\;}{\to }\;\;\left(n-1\right)\times 80S-\text{mRNA}+40S+60S \end{array}$$

$$\begin{array}{c} 1\times 80S-\text{mRNA}\;\overset{\;k_{3}\;}{\to }\;\text{mRNA}+40S+60S \end{array}$$

$$40S+60S\;\overset{\;k_{4}\;}{\to }\;80S$$

$$80S\;\overset{\;k_{5}\;}{\to }\;40S+60S$$

We assumed that the formation of inactive 80S is from the reversible assembly of free 40S and 60S subunits. Under sodium arsenite–treated conditions, we postulated that $k_{2}$ decreases to release free mRNA, triggering the formation of SGs. Here, we set free mRNA and G3BP1 as the drivers for the formation of SGs, which we explain in the following. In addition, $k_{4}$ increases, promoting the formation of inactive 80S ribosomes without mRNA. In the phase-field model, the concentration field of the i th component, including ribosomal components, eIFs, mRNA, and G3BP1, obeys the following dynamics

$$\frac{\partial c_{i}}{\partial t}=-\nabla \cdot \left(c_{i}v_{i}\right)+J_{i}$$

$$v_{i}=-\frac{k_{B}T}{\zeta_{i}}\nabla \left[\text{ln}c_{i}+\lambda_{i}c_{i}^{2}-C\nabla^{2}c_{i}+\chi_{\mathrm{ij}}c_{j}\right]$$

Here, $J_{i}$ is the chemical reaction flux that obeys the law of mass action for the i th component. $\zeta_{i}$ is the friction coefficient, C is the surface tension constant, $\chi_{\mathrm{ij}}$ is the interaction matrix, and the term $\lambda_{i}c_{i}^{2}$ is added to avoid large concentrations.

We set the energy unit as $k_{B}T$ , where $k_{B}$ is the Boltzmann constant, and T is the temperature. We set the time unit as 1 s, and we estimate $k_{3}$ as the inverse of the time for a ribosome to translate a typical protein (*61*, *62*), from which we obtain $k_{3}\approx 0.012$ . For simplicity, we set $\zeta_{i}$ to 1 for all components. Given the diffusion constant $D_{i}$ of ribosomal subunits as $0.31\;\mu m^{2}/s$ (*63*) and $D_{i}=k_{B}T/\zeta_{i}$ , we obtain the length unit as 0.55 μm.

The numerical simulations were performed in a 3D 31by31by31 grid by solving the model using the explicit Euler method with a periodic boundary condition on MATLAB. We set the grid size as 1 , the time interval for the simulations as 0.005 , the surface tension constant C=0.1 , and the number of ribosomes within a polysome to be less than 5 . We set λ=0.025 for G3BP1 and mRNA and $\chi_{G3\text{BP}1,\text{mRNA}}=-0.7$ and $\chi_{G3\text{BP}1,G3\text{BP}1}=-0.3$ as the major attractive interaction to generate phase separation. We also set λ=0.012 for ribosomal components, $\chi_{G3\text{BP}1,40S}=-0.039$ and $\chi_{G3\text{BP}1,\text{PIC}}=-0.090$ to increase the concentrations of 40S and PIC inside the condensates, and $\chi_{G3\text{BP}1,60S}=0.138$ and $\chi_{G3\text{BP}1,80S}=0.217$ to decrease the concentration of 60S and 80S inside the condensates. All the other elements of the interaction matrix were zero.

We set the reaction rate parameters as follows: $k_{1}=0.07$ , $k_{2}=5$ , $k_{3}=0.012$ , $k_{4}=0.008$ , and $k_{5}=0.002$ . At time t=500 , we mimicked the stressed condition by changing $k_{2}$ to $4\times 10^{-4}$ and $k_{4}$ to 0.02 . Meanwhile, we added a spatially uniform ±0.003 noise to $k_{3}$ to avoid deterministic dynamics and trigger phase separation. A minimum concentration $c_{\text{min}}=0.01\;\mu$ M for all components was set, below which the corresponding chemical reactions stop. The initial concentration of G3BP1 was 10μ M (*14*), while the initial concentrations of other components were based on the experimental data.

## Supplementary Materials

### The PDF file includes:

Figs. S1 to S9 Legends for movies S1 and S2 Legends for datasets S1 to S3

### Other Supplementary Material for this manuscript includes the following:

Movies S1 and S2

Datasets S1 to S3
