# Supplementary material for: Translation landscape of stress granules
Source: Sci Adv. 2025 Oct 3;11(40):eady6859. doi: 10.1126/sciadv.ady6859 (PMC12494010; doi:10.1126/sciadv.ady6859)
Supplement: Supplementary file 1 — Figs. S1 to S9 Legends for movies S1 and S2 Legends for datasets S1 to S3 [file sciadv.ady6859_sm.pdf]

Science Advances
AAAS

Supplementary Materials for

Translation landscape of stress granules

Yichun Wu *et al.*

Corresponding author: Jie Lin, linjie@pku.edu.cn; Qiang Guo, guo.qiang@pku.edu.cn

Sci. Adv. **11**, eady6859 (2025)
DOI: 10.1126/sciadv.ady6859

The PDF file includes:

Figs. S1 to S9
Legends for movies S1 and S2
Legends for datasets S1 to S3

Other Supplementary Material for this manuscript includes the following:

Movies S1 and S2
Datasets S1 to S3

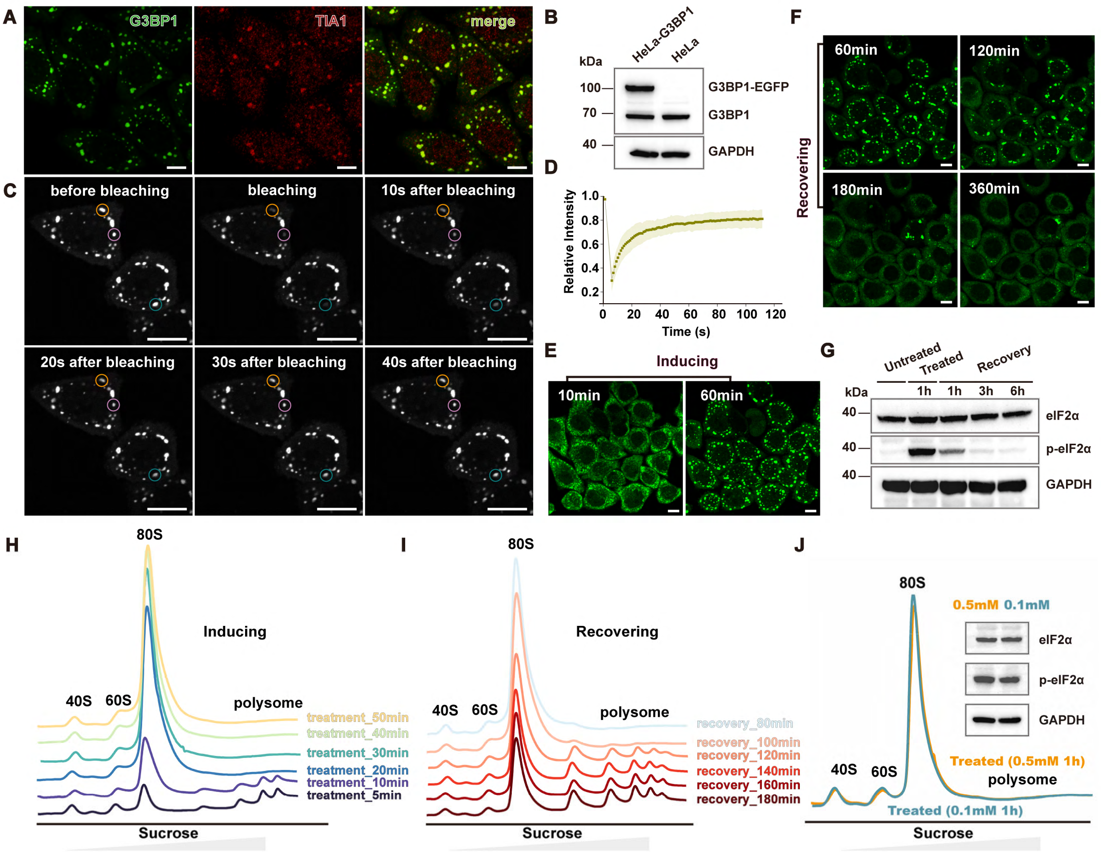

Fig. S1: Characterization and Dynamic Analysis of Stress Granules in HeLa Cells Expressing G3BP1-EGFP. (A) Immunofluorescence analysis of HeLa-G3BP1-EGFP cells following 1 hour of 0.5 mM sodium arsenite treatment. The image demonstrates the formation of stress granules (SGs), identified by both inherent G3BP1 fluorescence (left panel) and TIA1 immunostaining (middle panel). **(B)** Western blot analysis confirms the expression level of G3BP1-EGFP in the stable cell line. **(C)** Photobleaching experiment with confocal images captured at various time points (pre-bleach, bleach, and 10, 20, 30, and 40 seconds post-bleach). Three representative SGs, highlighted in orange, purple, and blue, were selected for photobleaching. **(D)** Average fluorescence recovery curve for SGs. Thirteen SGs were used for this analysis. The data points represent the average intensities, and the shaded areas represent the standard deviation. **(E)** Confocal microscopy images of SGs at different time points following treatment with sodium arsenite. **(F)** Confocal microscopy images of SGs at different time points following removal of the sodium arsenite stimulus. **(G)** Western blot results depict eIF2α protein levels and its phosphorylated form at various time points: untreated, 1-hour treatment, and recovery periods at 1, 3, and 6 hours. **(H)** Analysis of ribosomal components at consecutive time points (5, 10, 20, 30, 40, 50 minutes) following SG induction, as assessed by ribosome profiling. **(I)** Analysis of ribosomal components at consecutive time points (80, 100, 120, 140, 160, 180 minutes) during the gradual disappearance of SGs after stimulus removal, as assessed by ribosome profiling. **(J)** Ribosome profiling of cells treated with 0.5 mM and 0.1 mM sodium arsenite. Western blot analysis was performed to assess the phosphorylation levels under both conditions. Scale bar: 10 µm in **A**, 20 µm in **C**, 10 µm in **E** and **F**.

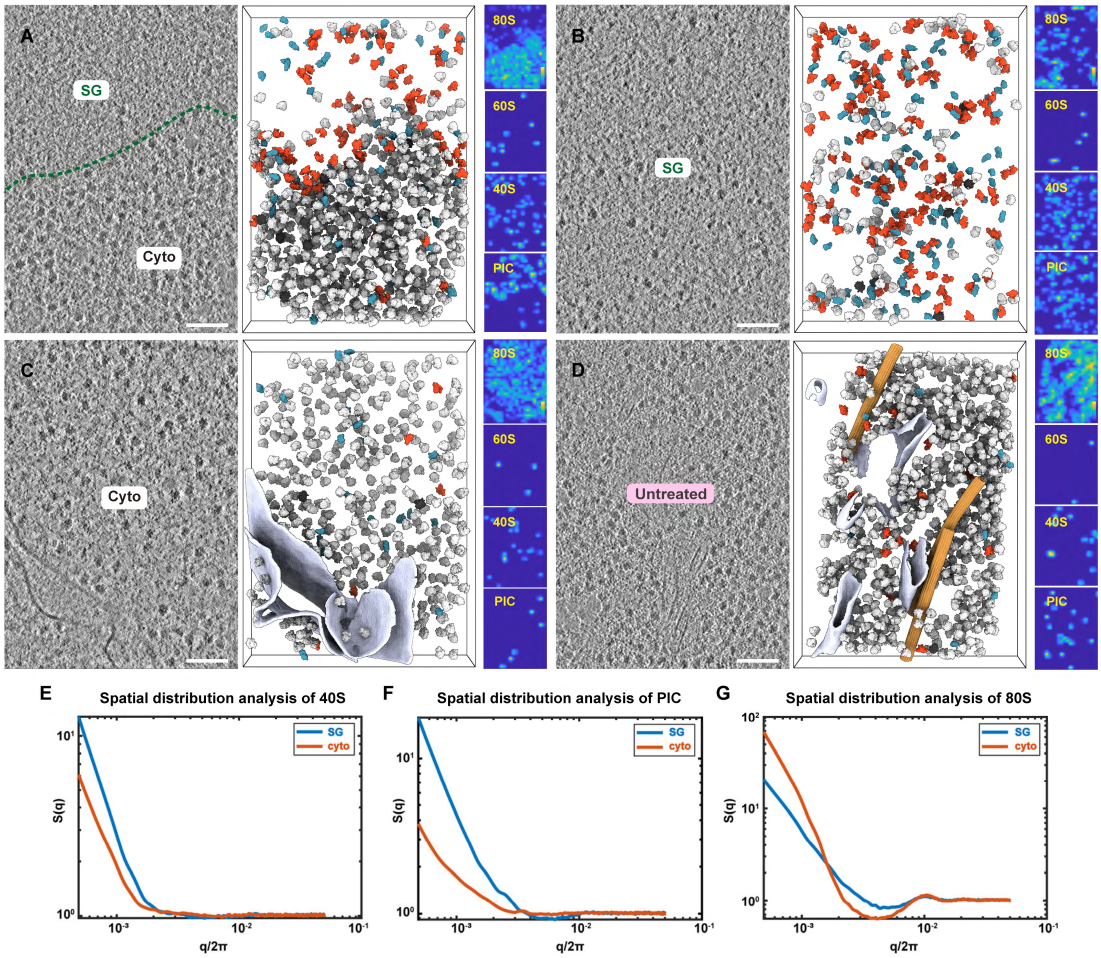

Fig. S2: Spatial Distribution Analysis of Ribosome Components. (A-D) Tomographic slices and 3D renderings of different regions in treated (**A**-**C**: SG boundary, SG, and cytoplasm) and untreated (**D**: cytoplasm) HeLa cells. The SG boundary was indicated by the green dashed line. Ribosomal components were placed back in their original orientations and locations, including 40S (blue), PICs (orange), 60S (dark grey) and 80S (grey). Microtubules (yellow), and ER membrane (light blue) are also segmented. After repositioning the particles, the voxel grey values were projected onto a 2D plane to generate heatmaps for each ribosomal component, with warmer colors (yellow) indicating higher local concentrations. **(E-G)** Charts illustrate the distribution characteristics of ribosomal components within and outside SGs based on the averaged structure factor across tomograms. The horizontal axis represents the wave number q divided by 2π, which corresponding to the reciprocal of the inter-particle distance. The vertical axis represents the structure factor $S(q)$, where $S(q)$=1 indicates randomly distributed particles (The increase of $S(q)$ at small wave numbers is attributed to the finite-size effect). Scale bar: 100 nm in **A**-**D**.

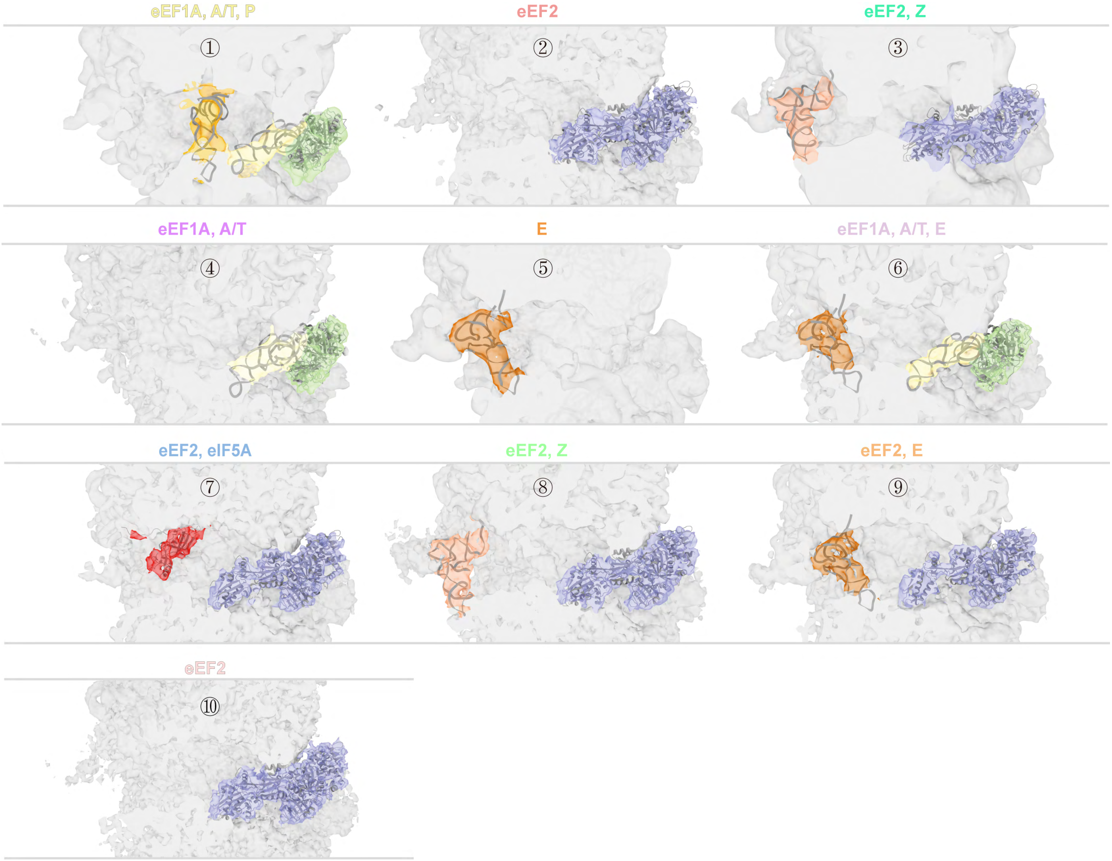

Fig. S3: Structural Validation of 80S Ribosome States. The density maps of 80S ribosome states corresponding to **Fig. 2** are displayed as transparent surfaces. The ribosome region is colored in grey, while the tRNAs and translation factors are color-coded as follows: A-site, P-site, E-site, and Z-site tRNAs are shown in light yellow, dark yellow, orange, and soft orange, respectively; eEF1A in green, eEF2 in purple, and eIF5A in red. Atomic models of the tRNAs and translation factors are superimposed for validation (PDB: 5lzs, 6z6m, 5gak).

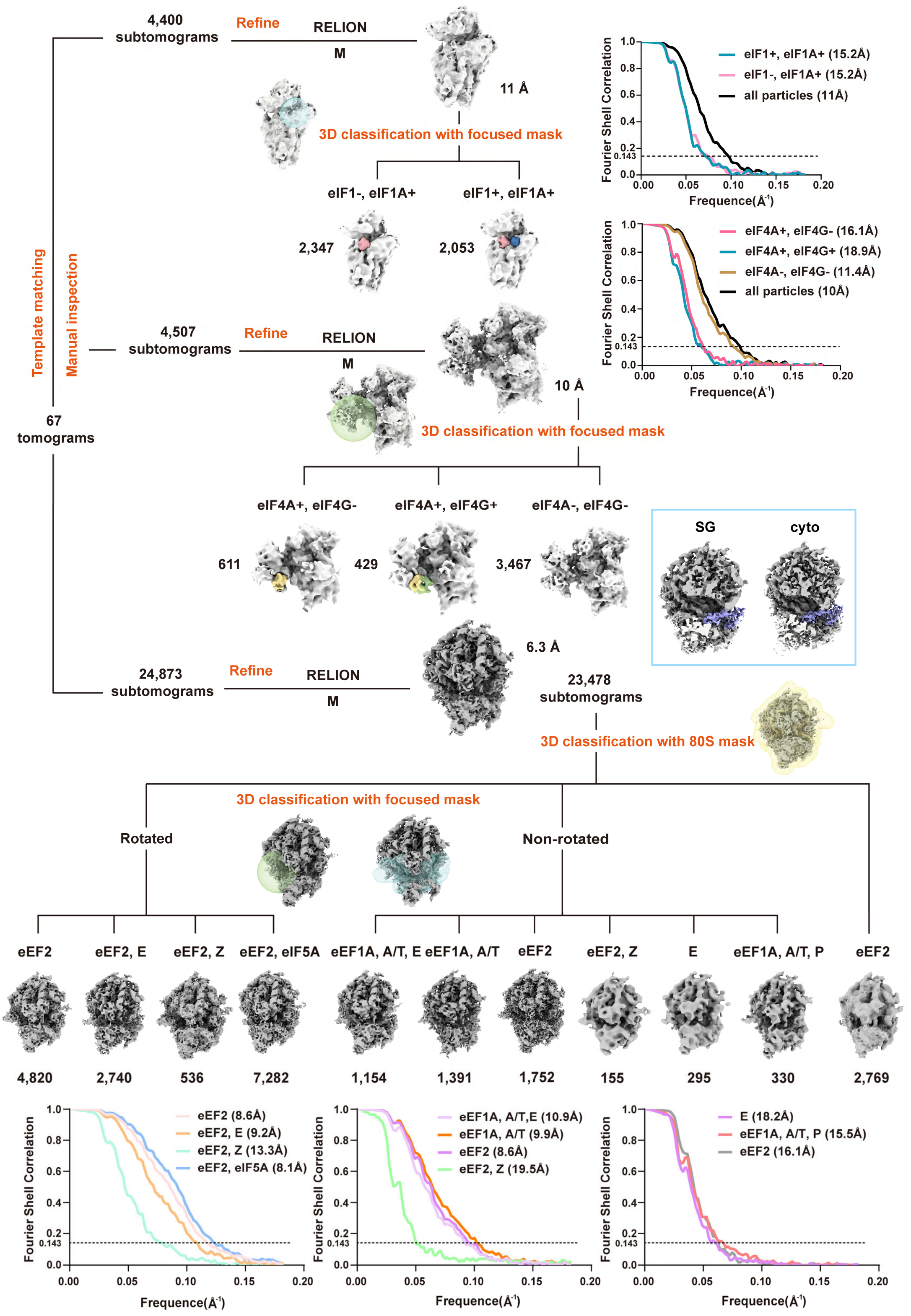

Fig. S4: Cryo-ET Data Analysis Workflow for HeLa Cells Under Sodium Arsenite Stress. Template matching was performed in STOPGAP initially. Subsequently, manual screening was performed to select the correct particles, resulting in a total of 4,400 40S subunits, 4,507 PICs, and 24,873 80S ribosomes for further analysis with RELION and M. After polishing the tomograms using M, the particles were re-extracted for classification in RELION. Using the eIF1- mask, the 40S subunits were classified into two states: eIF1A-only, eIF1 and eIF1A. With the eIF4 mask, three states were distinguished: eIF4A-only,

eIF4A and eIF4G, and neither eIF4A nor eIF4G. The 80S mask was utilized to distinguish between rotated and non-rotated states of the 80S ribosome, while the other two masks were employed for the classification of different factors and tRNA on the ribosome. Final classes were refined in RELION. FSC curves of corresponding ribosomal component states and their resolutions were estimated (FSC = 0.143). In the blue boxed region, the density maps of 80S ribosome generated using particles from SG and cytoplasm regions are presented side by side, showing their structural consistency.

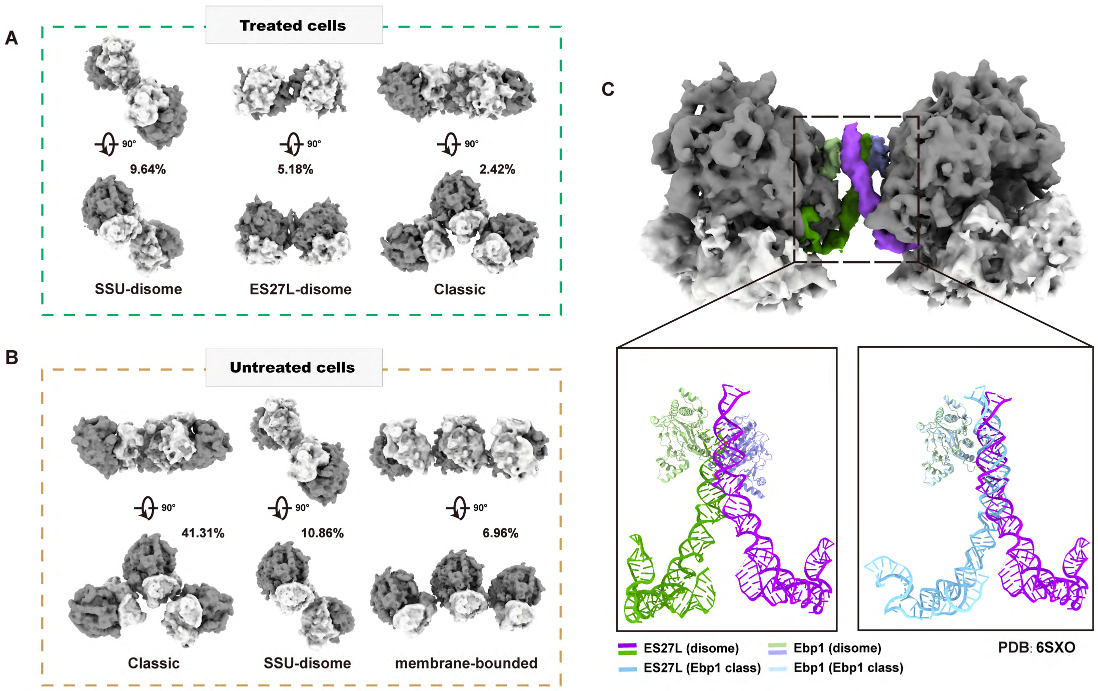

Fig. S5: Neighboring Topological Analysis of 80S Ribosomes in Sodium Arsenite-Treated and Untreated Cells. (A) Neighboring topological analysis of 80S ribosomes from sodium arsenite-treated cells identifies three distinct ribosomal clusters: SSU-disome, ES27L-disome, and Classic. These clusters represent 9.64%, 5.18%, and 2.42% of the total population, respectively. The large and small ribosomal subunits are depicted in dark and light grey, respectively. **(B)** In untreated cells, the same analysis identifies three major ribosomal clusters: Classic, SSU-disome, and membrane-bounded. These clusters constitute 41.31%, 10.86%, and 6.96% of the total population, respectively. The large and small ribosomal subunits are depicted in dark and light grey, respectively. **(C)** Schematic representation of the ES27L-disome. The large and small ribosomal subunits are depicted in dark and light grey. The density maps and models of Ebp1 and ES27L from the first ribosome are highlighted in green, while those from the second ribosome are shown in purple. A comparative view of Ebp1 and ES27L of mono-ribosome (blue, PDB: 6SXO) and a ES27L-disome (purple, this work) is also shown.

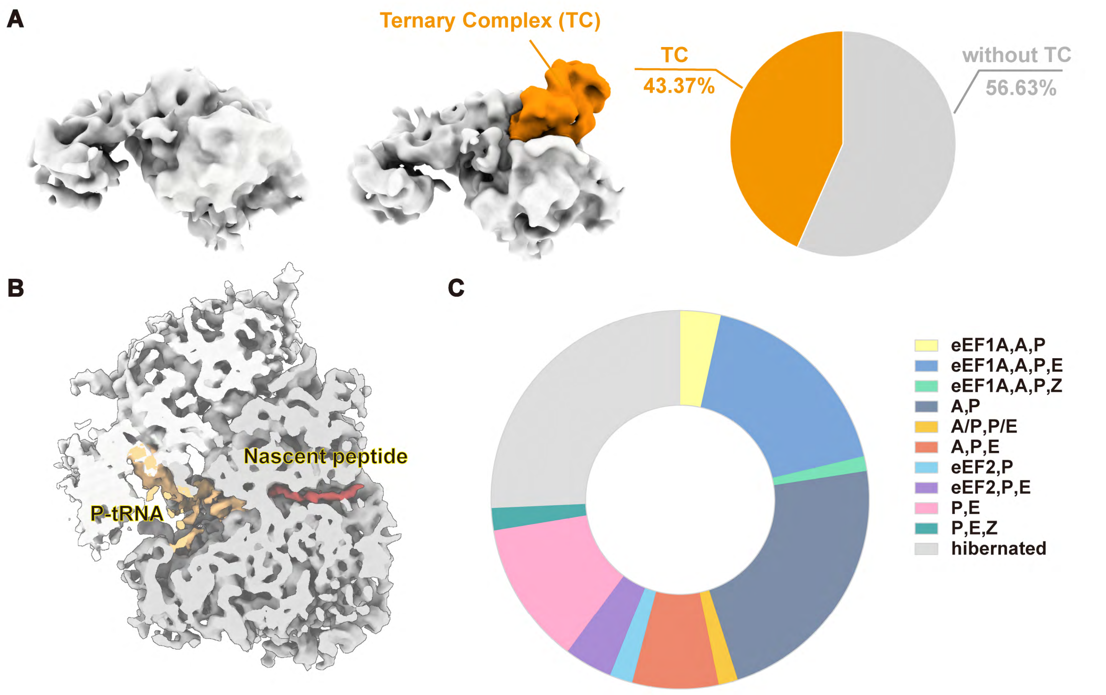

Fig. S6: Translation landscape in HeLa Cells without Stress. (A) Structural analysis of pre-initiation complex (PIC). The density map of PIC is shown in solid surface. Two different confirmations were identified after classification with population distribution shown as pie chart. The density corresponding to ternary complex was colored in orange. **(B)** Cross-sectional view of the directly reconstructed 80S ribosomes illustrates an active translating state, as demonstrated by the nascent peptide chain and the tRNA bound at the P site. **(C)** Pie chart displaying the relative proportions of 80S ribosome states identified in the untreated cells, with corresponding states color-coded and labeled alongside the chart.

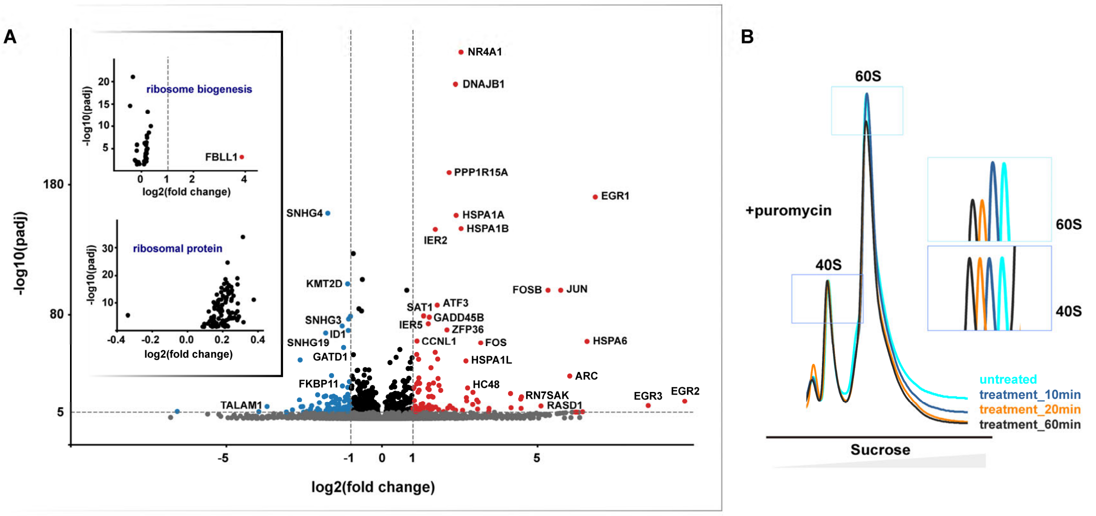

Fig. S7: Comprehensive Analysis of Ribosomal Responses to Sodium Arsenite Stress. (A) Volcano plot displaying RNA-seq results comparing HeLa cells treated with sodium arsenite for 1 hour to untreated cells. Significantly upregulated genes are highlighted in red, downregulated genes in blue, and genes with no significant change in black. Insets provide detailed analysis of genes associated with ribosome biogenesis pathways (top) and ribosomal proteins (bottom). **(B)** Ribosome profiling to assess the distribution of 40S and 60S ribosomal subunits at various time points (10 min, 20 min, 60 min) following sodium arsenite treatment, compared to untreated cells. To improve clarity, separate insets are used to display the distributions of 40S and 60S subunits, with the x-axis shifted to distinctly emphasize the distributions at each time point. Corresponding to **Fig. 3E**.

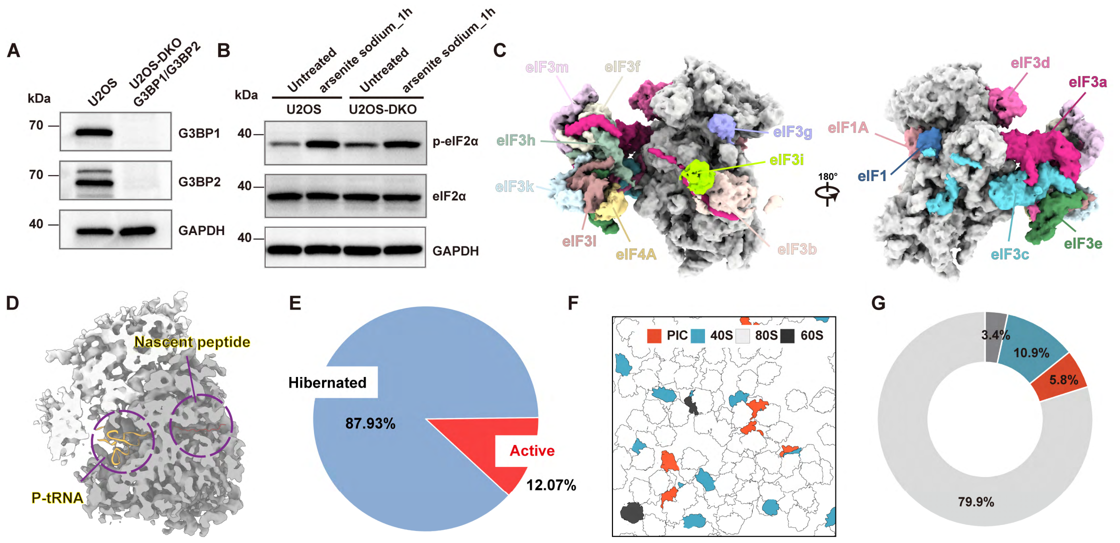

Fig. S8: Structural Analysis of Ribosomal Components in G3BP1/G3BP2 Double Knockout U2OS Cells under Sodium Arsenite Stress. **(A)** Western blot analysis comfirming the knockout of G3BP1 and G3BP2 in U2OS cells. **(B)** Western blot analysis showing the level of eIF2α and its phosphorylated form (p-eIF2α) in untreated and sodium arsenite-treated U2OS cells, both in wild-type and G3BP1/G3BP2 double knockout cells. **(C)** The density map of the pre-initiation complex (PIC) is shown in solid surface with two different views. Densities corresponding to initiation factors (eIF1, eIF1A, eIF4A, and subunits of eIF3) are colored according to **Fig. 2B**. **(D)** The cross-sectional view of the density map shows the overall structure of 80S ribosomes directly reconstructed under sodium arsenite treatment, revealing an empty P site and nascent peptide tunnel, indicating a non-translating ribosome. The 40S small subunit (SSU) is colored light grey, and the 60S large subunit (LSU) is colored dark grey. **(E)** Pie chart shows the relative abundance of ribosomes in active (red) and hibernated (blue) states, with the corresponding percentages indicated. **(F)** Representative 3D rendering showing the distribution of ribosomes in the cytoplasmic region of G3BP1/G3BP2 double knockout cells. **(G)** Donut chart shows the proportion of 40S subunits (blue), pre-initiation complexes (PICs, orange), 60S subuints (dark grey), and 80S ribosomes (light grey) in G3BP1/G3BP2 double knockout cells.

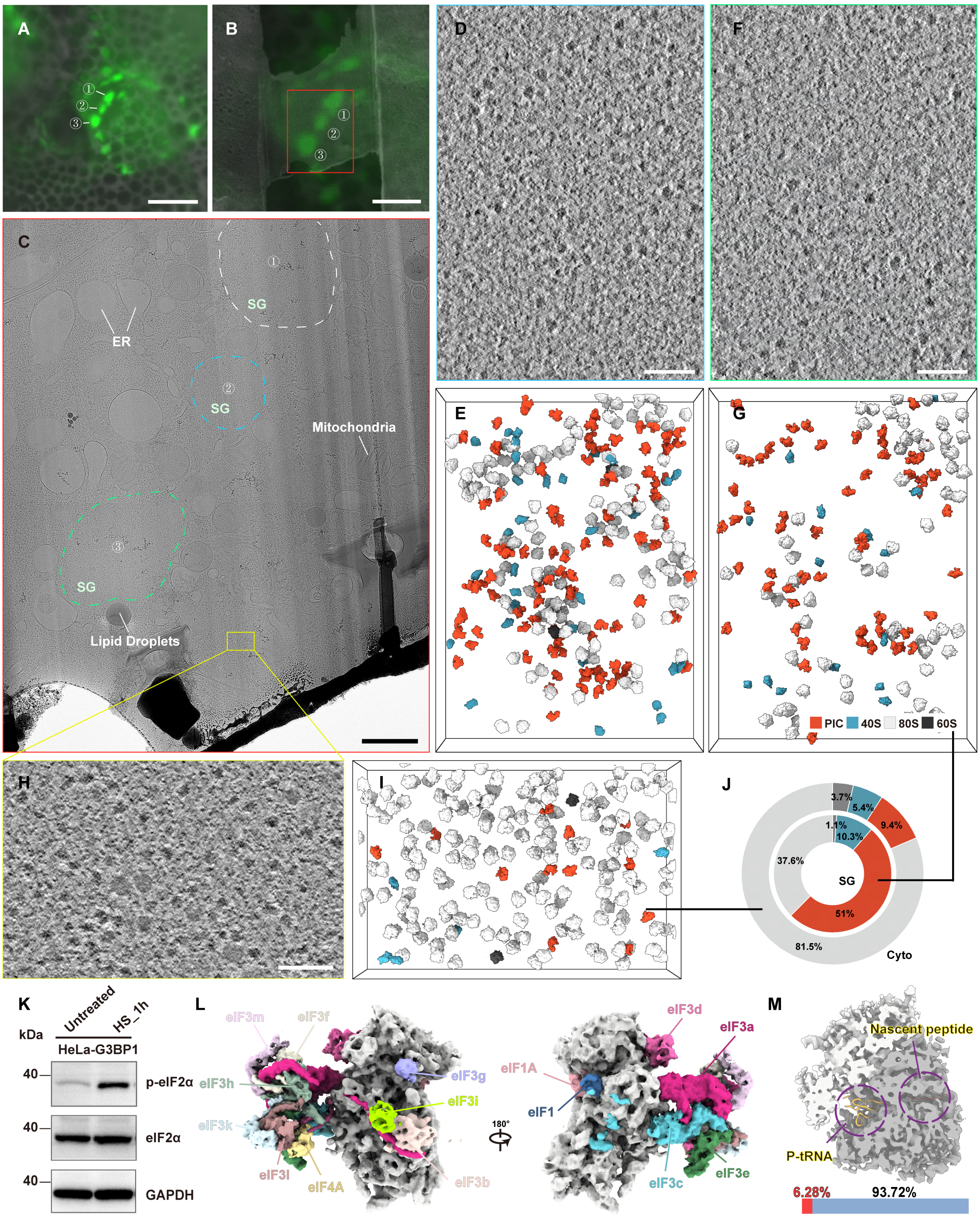

Fig. S9: In Situ Analysis of Heatshock-Induced Stress Granules in HeLa Cells. (A) A cryo-light microscopy image of HeLa cells following heatshock treatment. Stress granules are labeled with EGFP-G3BP1 (green). Three distinct stress granules are annotated with numbers for identification. **(B)** A superimposed light microscopy and scanning electron microscopy (SEM) image of the prepared lamella. The three annotated stress granules from panel **A** are represented in the lamella. **(C)** Transmission electron microscopy (TEM) image of the prepared lamella corresponding to panels **A** and **B**, with dashed lines outlining the approximate boundaries of the stress granules. Cellular structures such as the endoplasmic reticulum, mitochondria, and lipid droplets are also marked. **(D)** A tomographic slice corresponding to the portion of the stress granule outlined by the blue dashed line in panel **C**. **(E)** 3D rendering of the tomogram of panel **D**. Ribosomal components, including 40S ribosomes (blue), 60S ribosomes (dark grey), pre-initiation complexes (PICs, orange), and 80S ribosomes (grey), were

computationally identified and placed back into their original locations and orientations. **(F)** A tomographic slice corresponding to the portion of the stress granule outlined by the green dashed line in panel **C**. **(G)** 3D rendering of the tomogram of panel **F**. **(H)** A tomographic slice displaying the cytoplasmic region, highlighted by a yellow box in panel **C**. **(I)** 3D rendering of the tomogram of panel **H**. **(J)** The relative abundance of 40S subunits (blue), pre-initiation complexes (PICs, orange), 60S subunits (dark grey), and 80S ribosomes (light grey) inside and outside stress granules in heatshock-treated HeLa cells is depicted in a donut chart. **(K)** Western blot analysis showing the levels of eIF2α and its phosphorylated form in untreated and heatshock-treated conditions. **(L)** The density map of the pre-initiation complex (PIC) is shown in solid surface with two different views. Densities corresponding to initiation factors (eIF1, eIF1A, eIF4A, and subunits of eIF3) are colored according to **Fig. 2B**. **(M)** The cross-sectional view of the density map shows the overall structure of 80S ribosomes directly reconstructed under heatshock treatment, revealing an empty P site and nascent peptide tunnel, indicating a non-translating ribosome. The 40S small subunit (SSU) is colored light grey, and the 60S large subunit (LSU) is colored dark grey. A bar chart below the density map shows the relative abundance of ribosomes in active (red) and hibernated (blue) states, with the corresponding percentages indicated. Scale bar: 10 μm in **A**, 5 μm in **B**, 1 μm in **C**, and 100 nm in **D**, **F**, and **H**.

Supplementary Video 1

This supplementary video depicts the dynamics of stress granule formation in HeLa-G3BP1 cells treated with sodium arsenite. Imaging was performed at 2-minute intervals for one hour, commencing 6 minutes post-treatment. The video clearly demonstrates that stress granules begin to form at approximately 10 minutes, progressively growing to micrometer sizes by the end of the observation period.

Supplementary Video 2

This supplementary video illustrates the process of stress granule dissolution in HeLa-G3BP1 cells following the removal of sodium arsenite. Imaging commenced 6 minutes after drug withdrawal and was conducted at 2-minute intervals for a total duration of 6.5 hours. The video shows a gradual decrease in stress granule size, with most granules disappearing within 2 to 3 hours.

Supplementary Source Data 1

Source numerical data of Figure 2. Classification of 80S ribosomes into 11 conformational states showing particle number and relative proportions.

Supplementary Source Data 2

Source numerical data of Figure 3. Spatial distribution analysis of ribosomal components: tomogram-derived statistics and raw experimental measurements.

Supplementary Source Data 3

Source numerical data of Figure 4. Particle numbers of ribosomal components in different regions simulated by phase-based model.
